# Phytochrome A and B Regulate Primary Metabolism in *Arabidopsis* Leaves in Response to Light

**DOI:** 10.3389/fpls.2017.01394

**Published:** 2017-08-09

**Authors:** Xiaozhen Han, Takayuki Tohge, Pierce Lalor, Peter Dockery, Nicholas Devaney, Alberto A. Esteves-Ferreira, Alisdair R. Fernie, Ronan Sulpice

**Affiliations:** ^1^Plant Systems Biology Research Lab, Plant and AgriBiosciences, School of Natural Sciences, NUI Galway Galway, Ireland; ^2^Max Planck Institute of Molecular Plant Physiology Potsdam-Golm, Germany; ^3^Centre for Microscopy and Imaging, Anatomy, School of Medicine, NUI Galway Galway, Ireland; ^4^Applied Optics Group, School of Physics, NUI Galway Galway, Ireland

**Keywords:** phytochrome A, phytochromes B, chloroplast ultrastructure, starch accumulation, metabolites, light spectral content, light intensity

## Abstract

Primary metabolism is closely linked to plant productivity and quality. Thus, a better understanding of the regulation of primary metabolism by photoreceptors has profound implications for agricultural practices and management. This study aims at identifying the role of light signaling in the regulation of primary metabolism, with an emphasis on starch. We first screened seven cryptochromes and phytochromes mutants for starch phenotype. The *phyAB* mutant showed impairment in starch accumulation while its biomass, chlorophyll fluorescence parameters, and leaf anatomy were unaffected, this deficiency being present over the whole vegetative growth period. Mutation of plastidial nucleoside diphosphate kinase-2 (NDPK2), acting downstream of phytochromes, also caused a deficit in starch accumulation. Besides, the *glucose-1-phosphate adenylyltransferase small subunit (APS1)* was down-regulated in *phyAB*. Those results suggest that PHYAB affect starch accumulation through NDPK2 and APS1. Then, we determined changes in starch and primary metabolites in single *phyA*, single *phyB*, double *phyAB* grown in light conditions differing in light intensity and/or light spectral content. PHYA is involved in starch accumulation in all the examined light conditions, whereas PHYB only exhibits a role under low light intensity (44 ± 1 μmol m^-2^ s^-1^) or low R:FR (11.8 ± 0.6). PCA analysis of the metabolic profiles in the mutants and wild type (WT) suggested that PHYB acts as a major regulator of the leaf metabolic status in response to light intensity. Overall, we propose that PHYA and PHYB signaling play essential roles in the control of primary metabolism in *Arabidopsis* leaves in response to light.

## Introduction

Plants possess a number of photoreceptor proteins which perceive light signals and modify a myriad of physiological processes in plants (Briggs and Olney, 2001; Fankhauser and Staiger, 2002; Moglich et al., 2010). Phytochromes (PHYs) and cryptochromes (CRYs) are two major classes of photoreceptors which control over similar aspects of plant development such as de-etiolation, plant architecture and flowering (Franklin and Quail, 2010; Yu et al., 2010). PHYs absorb UV and blue and red and far-red, whereas CRYs sense only UV and blue (Lagarias and Rapoport, 1980; Cashmore et al., 1999; Chun et al., 2001; Usami et al., 2004; Castillon et al., 2009).

Phytochromes are formed of a chromophore and an apoprotein (Jones et al., 1986). They have two photoconvertible forms: the red absorbing form Pr and the far-red form Pfr (Lagarias and Rapoport, 1980). In *Arabidopsis*, there are five PHY isoforms, A, B, C, D, and E (Clack et al., 1994). Pfr form of PHYA is unstable, with a short half-life of 1–2 h while its Pr form is very stable with a half-life of ca. 1 week (Clough and Vierstra, 1997). PHYA protein accumulates to a high level in etiolated seedlings and acts primarily as a far-red sensor at the stages of early seedling development (Parks and Quail, 1993), whereas PHYB is the predominant red sensor (Reed et al., 1993; Franklin et al., 2003). Hypocotyl 1 (HY1) encodes a plastid heme oxygenase necessary for PHY chromophore biosynthesis, and deletion of HY1 leads to lowered levels of photoreversible PHY A, B, C, D, E, thus reduced sensitivity to far-red and red light (Chory et al., 1989; Muramoto et al., 1999).

Three genes encode for CRYs in *Arabidopsis* (Kleine et al., 2003). The cryptochrome 1 (*cry1*) mutant was the first identified due to its insensitivity to blue light dependent inhibition of hypocotyl elongation (Ahmad and Cashmore, 1993). This mutant also exhibits decreased anthocyanin levels, most likely due to reduced expression of anthocyanin biosynthetic enzymes (Ahmad et al., 1995). In contrast with the *cry1* mutant which shows an absence of inhibition of hypocotyl elongation across a wide range of blue light fluence rates, cryptochrome 2 (*cry2)* mutant exhibits such phenotype only under low blue light fluence. This is explained by high blue light intensity down-regulating the expression of *CRY2* gene as well as inducing degradation of the CRY2 protein (Ahmad et al., 1998; Lin et al., 1998).

Understanding the regulatory roles of PHYs and CRYs in primary metabolism, particularly carbohydrate metabolism, is of great importance for improving yield and quality of agricultural products under controlled light environments such as glasshouses (Darko et al., 2014). PHYA affect the levels of a series of primary metabolites including amino acids, organic acids and major sugars in response to far-red and white light in *Arabidopsis* rosettes (Jumtee et al., 2008). Light intensity can modify the contents of a large number of primary metabolites in *Arabidopsis* leaves (Florez-Sarasa et al., 2012). Recently, the over-accumulation of a large number of primary metabolites has been observed in the leaves of the *Arabidopsis phyBD* and *phyABDE* mutants (Yang et al., 2016). Besides, a number of studies have also demonstrated the involvement of PHYs and CRYs in regulating different traits directly linked with carbon assimilation such as leaf anatomy (Saebo et al., 1995; Yano and Terashima, 2001; Mao et al., 2005; Boccalandro et al., 2009) and photosynthetic machinery (Ahmad et al., 1995; Barneche et al., 2006; Toledo-Ortiz et al., 2014; Gururani et al., 2015). Recently, a set of genes encoding for starch synthetic enzymes has been found to be induced by PHYA under far-red (Chen et al., 2014). It is in contrast to an increased starch accumulation in *phyBD* and *phyABDE* (Yang et al., 2016).

Emerging evidences have suggested PHYs and CRYs signaling have impacts on the levels of primary metabolites. However, there are multiple isoforms of PHYs and CRYs and the discrepancies in light sources and light treatments used in laboratories could lead to different results. Therefore, more investigations are required to gain a comprehensive understanding about the regulation. Starch is the major source of carbon at night for *Arabidopsis* plants, and its metabolism is tightly linked to the whole primary metabolism (Sulpice et al., 2009). Here, we firstly performed a starch phenotype screen among seven *phys* or *crys* mutants. *phyAB* but not *cry1* and *cry2* showed impairment in starch accumulation despite the absence of a growth phenotype. Then we investigated the changes in starch and other primary metabolites in single *phyA*, single *phyB* and double *phyAB* compared with WT by growing the plants under light conditions varying in light intensity and light spectral content. The results suggest that PHYA and PHYB signaling play important roles in the regulation of starch and many other primary metabolisms in plant leaves, being influenced by not only light intensity but also light spectral content.

## Materials and Methods

### Plant Material and Growth Conditions

Nine *Arabidopsis thaliana* photoreceptor mutants were used in this study including *phyAB, phyA, phyB, hy1, hy1/cry1, hy1/cry2, hy1/cry1/cry2, cry1*, and *cry2*. They were obtained from NASC, and their detailed information is provided in Supplementary Table 1. Dry and unstratified seeds were directly sown in 4-cm-diameter pots (3 seeds/pot, one plant were kept) filled with soil (peat, perlite, and vermiculite: 5:1:1) in growth chamber with a 16 h light/8 h dark cycle, at 20°C/16°C (day/night) and 60%/75% humidity (day/night). Pots of different lines were randomized to minimize positional effects. No fertilizer was applied during the growth. Unless otherwise stated, plants were harvested 20 days after sowing and before bolting. Independent samples, each containing two rosettes, were harvested within the last hour of the day (ED), or night (EN). Harvested rosettes were immediately put into liquid nitrogen, and stored at -80°C until use. For extractions, the leaves were pulverized with liquid nitrogen to a fine powder using a tissue lyser (Qiagen, Hilden, Germany).

As white light sources, two sets of fluorescent lamps (FLs) with contrasted spectral quality were used: FL1 (The Philips Master TL-D Reflex 58W/840) and FL2 (Philips Master TL-D 58W/840). Treatments with Red light (enriched between 570 nm and 720 nm) were obtained with FL1 covered with a red plastic Neewer^®^ 30 cm × 30 cm transparent color correction light gel filter similarly as in Reed et al. (1994). Light intensity was determined by use of a ‘Standard’ Fibre Optic Light Measuring System and PAR quantum sensor (Skye Instruments Ltd.). Light spectrum profiles were measured with a USB2000+ spectrometer (Ocean Optics). The spectral data for FL1, FL2, and Red light is presented in Supplementary Data Sheet 1 (Spectral datasets). The photostationary state of PHY (PSS) and yield photon flux (YPF) were calculated according to Sager et al. (1988). The red to far-red ratio (R:FR) was derived by dividing the total counts/photon flux from red light (600–700 nm) by that from far-red light (700–800 nm). To assess how light influences the individual role of PHYA and PHYB in primary metabolism, *phyA, phyB, phyAB*, and WT were grown under different light conditions varying in light spectral content and/or light intensity. The light characteristics of the different conditions are presented in **Table 1**. High (H) light intensity and low (L) light intensity were achieved by adjusting the distance of plants from the lamps.

**Table 1:** Characteristics of the light conditions for growing *phyA, phyB, phyAB*, and WT.

	Lamp sets	PPFD (μmol m^-2^ s^-1^) (400–700 nm)	YPF	R:FR	PSS
C1	FL1	251 ± 4	220 ± 4	11.8 ± 0.6	0.855 ± 0.001
C2	FL2	251 ± 4	222 ± 4	14.3 ± 0.2	0.864 ± 0.001
C3	FL1	136 ± 3	119 ± 3	11.8 ± 0.6	0.855 ± 0.001
C4	FL2	136 ± 3	120 ± 3	14.3 ± 0.2	0.864 ± 0.001
Red light	FL1	44 ± 1	43 ± 1	14.8	0.870

*Values were given as mean ± SE. Measurements were made at eight or nine different places where plants were grown. C1–C4 indicates white light conditions 1–4. C1 and C2, C3 and C4 differ in light spectral content, represented by R:FR and PSS (photostationary state of phytochrome); C1 and C3, C2 and C4 differ in light intensity, represented by PPFD and YPF (yield photon flux). Red light indicates red light condition.*

### Metabolite Analyses

The 10–20 mg of leaf powder was extracted three times with ethanol (250 μL 80% ethanol, 150 μL 80% ethanol, 250 μL 50% ethanol). Extracts were incubated for 20 min at 80°C at each step. After each extraction, the extracts were centrifuged at 14000 rpm for 5 min and the supernatants were transferred. The pellets from the last centrifugation were kept for starch determination. The combined supernatants were used to measure soluble sugars.

Starch pellets were boiled for 30 min in 400 μL 0.1 M NaOH at 95°C, and then neutralized with 80 μL 0.5 M HCl, 0.1 M acetate/NaOH, pH 4.9. The neutralized solution was digested overnight at 37°C with 100 μL starch degradation mix (7 U/ml amyloglucosidase + 12 U/ml amylase in 50 mM acetate buffer pH 4.9). Digested starch and soluble sugars were determined by enzymatic assay (Cross et al., 2006) using a microplate reader (BioTek GmbH, Germany).

Derivatization and gas chromatography-mass spectrometry analysis were performed as described previously (Lisec et al., 2006), starting from aliquots of 30 mg frozen FW.

### Chlorophyll Fluorescence Analysis

A PAM-2500 fluorometer (PAM-2500, Walz GmbH, Germany) was used according to manufacturer’s instructions. Leaves were firstly dark adapted for 20 min to determine the dark fluorescence yield (*F*_0_), and then a red saturation pulse (Int: 9) was applied for 5 s to determine *F*_m_. The built-in actinic red light (144 μmol photons m^-2^ s^-1^) was then turned on for around 10 min, followed by a saturation pulse to get *F*_m_′ when the momentary fluorescence yield *F* was stable. Chlorophyll fluorescence parameters were calculated according to Kitajima and Butler (1975), Genty et al. (1989), and Genty et al. (1996) as follows: *F*_v_/*F*_m_ = (*F*_m_ -*F*_0_)/*F*_m_; *Y*(II) = (*F*_m_′ -*F*)/*F*_m_′; *Y*(NPQ) = *F*/*F*_m_′ -*F*/*F*_m_; *Y*(NO) = *F*/*F*_m_. For each genotype, leaves from four individual plants were measured. *F*_v_/*F*_m_, *Y*(II), *Y*(NO), and *Y*(NPQ) provide values of the quantum yield for the maximal PSII, the effective PSII, the non-regulated heat dissipation and fluorescence emission and the regulated heat dissipation, respectively.

### Transmission Electron Microscopy

Leaf portions (0.5 cm × 0.5 cm) from middle parts of fully expanded mature leaves were harvested at midday and fixed immediately in 2% v/v formaldehyde and 2% v/v glutaraldehyde in 0.1 M sodium cacodylate buffer for 2 h at room temperature in a desiccator under vacuum. Samples were washed in 0.1 M sodium cacodylate buffer three times for 5 min each time. The samples were then post-fixed in 1% w/v osmium tetroxide followed by washing in 0.1 M sodium cacodylate buffer. After washing, samples were sequentially dehydrated in 30, 50, 70, 90, and 100% ethanol for 20 min each. Then samples were infiltrated with 30, 50, 70, 90, and 100% London Resin medium grade (Agar Scientific) on a rotating wheel for 2 h each step, the resin being diluted in ethanol. After that, the 100% resin was replaced with fresh 100% resin and the samples were kept left on the rotating wheel overnight. The resin was replaced again with fresh 100% resin and left on the rotating wheel at room temperature for a further 3 h. Finally, samples were polymerized in gelatin capsules at 60°C for 2 days.

Transverse sections (1 μm) containing mesophyll cells were stained with 1% (w/v) Toluidine Blue (in 1% [w/v] boric acid) and visualized with an Olympus BX51 light microscope equipped with a DP70 digital camera (Olympus). For thickness measurement, two leaf sections from different plants were investigated for each line. The thickness of each section was assessed at 10 positions. Ultrathin sections (60–80 nm) were collected on formvar carbon-coated copper grids. Samples were then stained with lead citrate and uranyl acetate. Transmission electron microscopy (Hitachi H-600) was used to examine the ultrastructure of chloroplasts. Ten images of chloroplasts were analyzed for each line to estimate the ultrastructural changes. ImageJ was used for the thickness and area measurements.

### Gene Expression

Total RNA was isolated from 20 mg of leaf powder using the ISOLATE II RNA mini Kit (Bioline) according to the manufacturer’s instructions. mRNA concentration was measured at 260 nm using a NanoDrop ND-1000 UV-Vis spectrophotometer (Nano-Drop Technologies, Böblingen, Germany). The 10 ng of mRNA sample was used for cDNA synthesis according to the manufacturer’s instructions using the SensiFAST^TM^ cDNA synthesis kit (Bioline). Primer pairs for quantitative RT-PCR experiments are listed in Supplementary Table 2. Primers for *granule-bound starch synthase 1 (GBSS1)* were as in Tenorio et al. (2003), *glucose-1-phosphate adenylyltransferase large subunit (APL1), glucose-1-phosphate adenylyltransferase small subunit (APS1), starch synthase 1 (SS1), SS2, and SS4* as in Pyl et al. (2012). Quantitative PCR was performed using the SensiMix SYBR No-ROX Kit (Bioline, United Kingdom) according to the manufacturer’s instructions. The thermal conditions for the amplification reaction and melt curve analysis set on the CFX96 machine (Bio-Rad, United States) were as follows: 95°C for 10 min followed by 40 cycles PCR (95°C, 15 s; 60°C, 30 s; 72°C, 30 s), 95°C for 15 s, 70°C to 95°C with an increment of 0.5°C within 10 s. Relative quantification was adopted in this study and *ACTIN2* was used as internal reference gene. Relative expression level was derived from the ratio between 2^cq^_ref_/2^cq^_target_ for each sample.

### Statistical Analyses

The results are presented as mean ± standard errors (SE). Significant differences among the means of multiple genotypes were compared by analysis of one-way ANOVA with *post hoc* Tukey HSD (Honestly Significant Difference) test at *P* < 0.05 using IBM SPSS Statistics 23.0 software. Different letters indicate statistically significant differences of means. Student’s *t*-tests (*P* < 0.05) were used for comparison of transcripts levels as well as starch content of plants during vegetative growth between *phyAB* and WT. Three-way ANOVA was performed in SPSS at *P* < 0.05 to determine if there are interaction effects among light spectral content, light intensity and genotype on metabolites levels. The PCA analysis was also performed using SPSS.

## Results

### Biomass, Chlorophyll Fluorescence Parameters, and Starch Phenotypes of the Photoreceptor Mutants Grown under White Light

The *phyAB* mutant had different leaf shape with longer petioles but no change in fresh biomass compared with WT (Supplementary Figure 1). In contrast, *hy1, hy1/cry2, hy1/cry1*, and *hy1/cry1/cry2* displayed green–yellow leaves and severely reduced leaf biomass (Supplementary Figure 1). The *cry1* mutant did not show any significant difference in its growth phenotype, while *cry2* had slightly higher fresh biomass (Supplementary Figure 1).

We next measured chlorophyll fluorescence parameters of photoreceptor mutants and WTs. *F*_v_/*F*_m_ was higher than 0.780 for all the lines indicating they did not suffer from stress (Supplementary Table 3). *Y*(II), which reflects the relative photosynthetic efficiency, was significantly decreased in the *hy1/cry1* and *hy1/cry1/cry2* mutants, but remained unchanged in other photoreceptor mutants when compared with their corresponding WTs (Supplementary Table 3). Only *hy1/cry1* and *hy1/cry1/cry2* mutants were significantly higher for *Y*(NPQ), which means more energy was dissipated by regulated heat dissipation in those mutants compared to their respective WTs (Supplementary Table 3). *Y*(NO), which indicates the light energy dissipated by non-regulated heat dissipation and fluorescence emission, did not show dramatic changes in any of the mutants with respect to their respective WTs (Supplementary Table 3).

**FIGURE 1:**
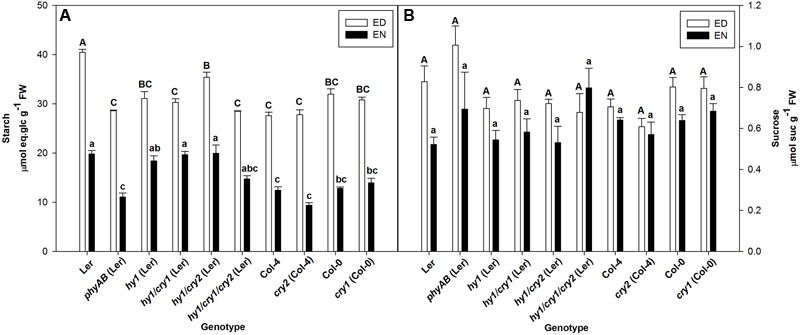
**(A,B)** Starch and sucrose contents at ED and EN in photoreceptor mutants and their respective WTs. Plants were grown under FL2 with PPFD of 115 ± 5 μmol m^-2^ s^-1^. Results are mean ± SE of measurements made on five biological replicates. Significant differences (*p* < 0.05) are indicated by different letters, uppercase for ED, and lowercase for EN.

Single *cry1* and single *cry2* mutants showed no difference with WT in starch accumulation at the end of the day (ED) and in their starch turnover, which is represented by the difference between starch at ED and EN (**Figure 1**). Double *phyAB* and single *hy1* mutants accumulated similar amounts of starch at ED, the amounts being noticeably lower than WT (**Figure 1**). The triple mutant *hy1/cry1/cry2* has a similar starch phenotype to single *hy1* mutant, which suggests that knockout of both CRY1 and CRY2 does not have any significant influence on starch accumulation or degradation (**Figure 1**). In agreement, starch accumulation in *hy1/cry1* and *hy1/cry2* was not significantly different from the single *hy1* mutant (**Figure 1**). However, starch accumulation in *hy1/cry2* mutant was significantly higher than in the *hy1/cry1* mutant (**Figure 1**).

Interestingly, the *phyAB* mutant did not show any clear impairment in starch degradation compared with WT because the decrease in starch accumulation at ED was accompanied by a similar decreased level of starch at EN (**Figure 1**).

Sucrose levels at ED and EN in all photoreceptor mutants were not different from their respective WTs (**Figure 1**). Glucose levels were also unaffected in the mutants, with the exception of *phyAB* which accumulated more glucose than WT at ED (Supplementary Table 4).

### Leaf Anatomy of *phyAB* and *hy1*

A deficit in starch accumulation was particularly prominent for *phyAB* and *hy1* mutants, so we investigated the leaf structure of the two mutants. *phyAB* showed no significant difference with Ler for leaf thickness, number of cell layers in palisade (2) and spongy mesophyll (3–4) (**Table 2** and Supplementary Figure 2A). By contrast, the leaves of the *hy1* mutant were significantly thinner than the WT, which can be explained by a reduced number of cell layers (**Table 2** and Supplementary Figure 2A). It was difficult to categorize the second cell layer in the *hy1* mutant as it had a structure between palisade mesophyll and spongy mesophyll (**Table 2** and Supplementary Figure 2A). In addition, in this mutant, the cell organization of the palisade mesophyll was relatively loose with more apoplastic space between cells compared with WT (**Table 2** and Supplementary Figure 2A).

**Table 2:** Characteristics of leaf anatomy and chloroplast ultrastructures in WT, *phyAB*, and *hy1* lines.

	Ler	*phyAB*	*hy1*
Leaf thickness (μm)	129.9 ± 2.5a	134.6 ± 2.0a	102.8 ± 1.7b
Palisade cell layer number	2	2	Intermediate cell type
Spongy cell layer number	3–4	3–4	Intermediate cell type
Palisade/spongy:	1.09 ± 0.03a	1.0 ± 0.03ab	Intermediate cell type
Chloroplast size (μm^2^):	16.1 ± 1.8a	16.8 ± 2.0a	11.5 ± 0.9b
Thylakoids/granum:	4 ± 0.5a	4.4 ± 0.7a	3.2 ± 0.3a
Plastoglobules/chloroplast:	34.8 ± 1.0a	12 ± 1.1c	20.7 ± 1.2b
Starch number/chloroplast:	3.6 ± 0.5a	3.3 ± 0.4a	3.0 ± 0.5a

*Plants were grown under FL2 with PPFD of 115 ± 5 μmol m^-*2*^ s^-*1*^. Results are mean ± SE of measurements made with 10 images of sections from two individual plants per genotype. Representative images are in Supplementary Figure 2. Numbers followed by different letters are significantly different (*p* < 0.05).*

Since starch resides in chloroplasts, we investigated possible modifications in the chloroplast ultrastructure of *phyAB* and *hy1* mutants by transmission electron microscopy. The *hy1* mutant had smaller chloroplasts (**Table 2** and Supplementary Figure 2B). Both *phyAB* and *hy1* mutants showed decreased numbers of plastoglobules per chloroplast, the decrease being particularly large in *phyAB* mutant (**Table 2** and Supplementary Figure 2B). The number of starch granules per chloroplast and thylakoids per granum did not vary from WT in *phyAB* and *hy1* mutants (**Table 2** and Supplementary Figure 2B).

The *hy1* mutant showed severe growth impairment with light green/yellow leaves and large modifications in its leaf structure, with thinner leaves, less cell layers and undefined palisade mesophyll, which could lead to confounding effects and thus a difficulty to interpret our starch results. Thus, we decided to avoid using this mutant for the next experiments.

### Starch Content in *phyAB* Mutant over Vegetative Development

**FIGURE 2:**
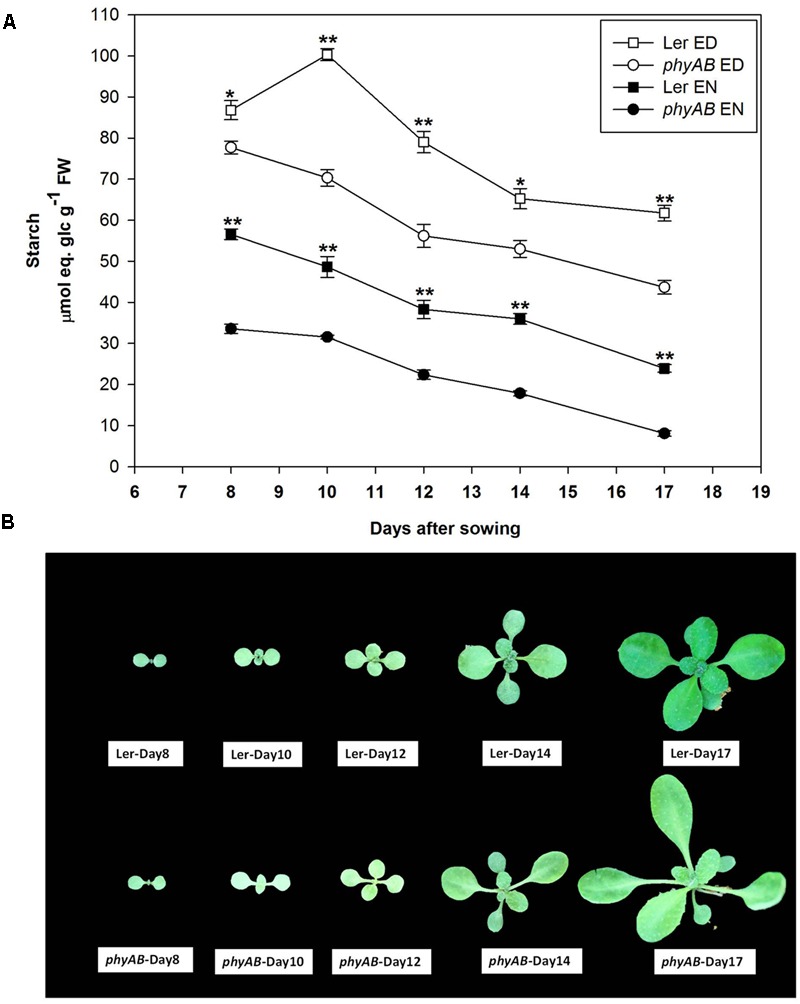
Starch content and morphology of *phyAB* and WT during the vegetative growth. **(A)** Starch contents at ED and EN in *phyAB* and WT. Results are mean ± SE of measurements made on three biological replicates. Asterisks indicate statistically significant differences of starch contents at ED or at EN between *phyAB* and WT (^∗^*P* < 0.05; ^∗∗^*P* < 0.01). **(B)** Pictures of WT and the *phyAB* mutant before harvest. Plants were grown under FL1 with PPFD of 125 ± 5 μmol m^-2^ s^-1^.

Given that PHYs regulate plant development, and that the amount of starch may vary during the growth of the plants, we next determined starch amounts in *phyAB* and WT during their vegetative growth from day 8 to day 17 after sowing. The *phyAB* mutant had consistently less starch than WT, both at ED and EN. Interestingly, its starch turnover was similar to WT during the development of the plants (**Figure 2**). The starch accumulated at ED decreased in both the mutant and WT while the plants aged, this decrease being concomitant to a decrease at EN (**Figure 2**). Further, the turnover of starch remained relatively stable over time in both WT and the mutant during vegetative growth (**Figure 2**).

### PHYAB Might Regulate Starch Accumulation through NDPK2 and APS1

Nucleoside diphosphate kinase 2 and PIFs are the primary signal transducers of PHYs (Choi et al., 1999; Castillon et al., 2007). Mutation of PIF4 does not lead to a deficit in starch accumulation (Mugford et al., 2014). However, the *ndpk2* mutant showed a starch deficit phenotype at ED and EN (**Table 3**). We also examined the diurnal transcript profiles of genes encoding starch synthetic enzymes in *phyAB*. Among them, only *APS1* showed significant differences in expression amplitude at one time point (ZT8) between *phyAB* and WT (Supplementary Figure 3).

**Table 3:** Chlorophyll fluorescence analysis and starch contents in *ndpk2* and WT.

	*F*_v_/*F*_m_	*Y*(II)	Starch (μmol eq. glc g^-1^ FW)	
			ED	EN
Col-0	0.787 ± 0.003a	0.345 ± 0.006a	40.0 ± 0.5a	12.2 ± 0.6a
*ndpk2*	0.790 ± 0.003a	0.341 ± 0.010a	32.1 ± 0.8b	8.8 ± 0.6b

*Plants were grown under FL2 with PPFD of 115 ± 5 μmol m^-*2*^ s^-*1*^. Results are mean ± SE of measurements made on three biological replicates. Numbers followed by different letters are significantly different (*p* < 0.05).*

### Starch and Sugar Contents in *phyA, phyB*, and *phyAB* under Red Light and Different White Light Conditions

**FIGURE 3:**
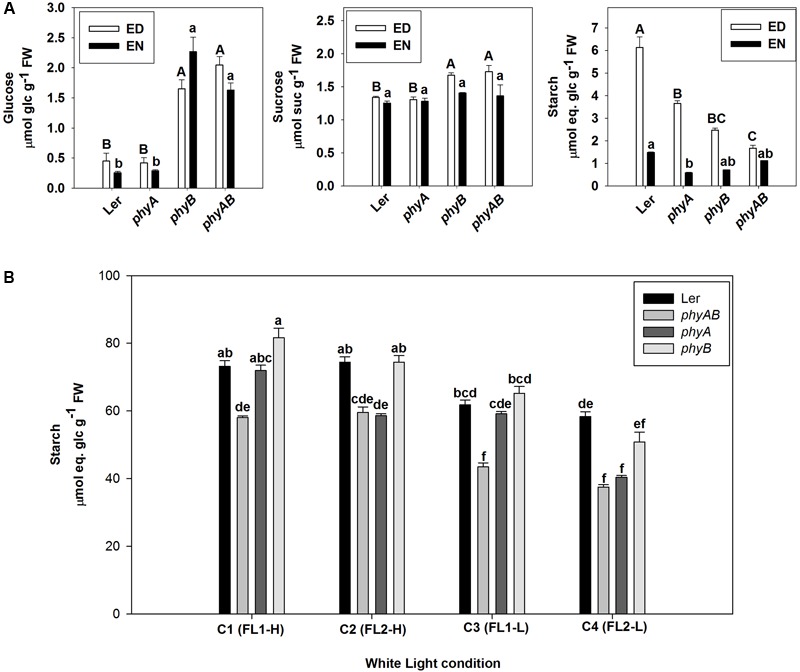
Starch and soluble sugar levels in *phyA, phyB, phyAB*, and WT grown in different light conditions. **(A)** Glucose, sucrose, and starch at ED and EN in plants grown under red light. Results are mean ± SE of measurements made on four biological replicates. **(B)** Starch at ED in plants grown under four white light conditions. Results are mean ± SE of measurements made on three biological replicates. Means of the genotype^∗^light condition population were compared by one-way ANOVA with *post hoc* Tukey HSD test at *P* < 0.05. Significant differences (*p* < 0.05) are indicated by different letters.

As PHYs are mainly responsive to red and far-red light (Lagarias and Rapoport, 1980), we then grew *phyA, phyB*, and *phyAB* and WT under red light. The mutants had significantly reduced starch accumulation at ED under red light compared with WT (**Figure 3A**). The largest decrease in starch accumulation was observed for *phyAB*, with *phyB* and *phyA* exhibiting intermediate starch accumulation (**Figure 3A**). In contrast, only *phyA* had significantly lower starch content at EN than WT, with *phyB* and *phyAB* showing equivalent contents to WT (**Figure 3A**). When plants were grown under red light, the levels of sucrose in *phyB* and *phyAB* at ED and glucose at both ED and EN were significantly higher than WT, while *phyA* did not show any significant difference (**Figure 3A**), suggesting a major role of PHYB.

As both PHYA and PHYB have a role in promoting starch accumulation under red light of 44 ± 1 μmol m^-2^ s^-1^, we then examined their roles in four white light conditions. WT plants accumulated similar amounts of starch when grown under the same light intensity (**Figure 3B**). There was significantly less starch accumulated at ED in *phyAB* mutant compared with WT under all of the four light conditions (**Figure 3B**). The *phyA* mutant accumulated similar amount of starch than *phyAB* when grown under C2 (FL2-H) and C4 (FL2-L), but accumulated the same level of starch as WT for C1 (FL1-H) and C3 (FL1-L) (**Figure 3B**). Thus, the *phyA* mutant showed a deficit in starch accumulation when growth light had qualitatively higher R:FR (14.3 ± 0.2) and PSS (0.864 ± 0.001) (**Figure 3B**). By contrast, the *phyB* mutant did not exhibit any difference in starch content from WT (**Figure 3B**). The whole experiment was repeated, and qualitatively, we observed the same results (Supplementary Table 5).

### Metabolite Levels in *phyA, phyB*, and *phyAB* under Different White Light Conditions

In order to find out if the changes in starch accumulation observed in those mutants grown in the four white light conditions were associated with a general modification of the primary metabolism, GC-MS profiles were obtained for the same leaf samples. Sixty-six compounds were quantified (Supplementary Table 4). To isolate the effects of genotype, light intensity, and spectral content on starch and other metabolites levels, we then performed a three way ANOVA (Supplementary Figure 4). Spectral content, light intensity, and genotype all have significant influences on starch accumulation individually, and genotype had interacting effects with both light intensity and spectral content on starch accumulation (Supplementary Figure 4). Among the 66 compounds detected by GC-MS, 31, 45, and 42 compounds showed significant dependence for light spectral content, light intensity and genotype, respectively (Supplementary Figure 4). Besides, spectral content, light intensity and genotype also had interactive effects for many compounds (Supplementary Figure 4). The genotype effect was markedly high for C1 (FL1-H), with 24 metabolites being significantly different in mutants compared to WT, while 11 in C2 (FL2-H), 9 in C3 (FL1-L), and 8 in C4 (FL2-L) were different from WT in the mutants (Supplementary Table 6).

In C1 (FL1-H), 10 metabolites involved in amino acids metabolism were down-regulated in *phyAB*, 13 in *phyA* and 8 in *phyB* compared with WT. Besides, erythritol and galactose, involved in carbohydrate metabolism, were significantly decreased in *phyAB* and *phyB* mutants in comparison with WT in C1 (FL1-H). The levels of threonate involved in redox regulation, and citrate and succinate involved in the glyoxylate and TCA cycles were lower in *phyAB* and *phyA* mutants than in WT in C1 (FL1-H). Ethanolamine and octadecanoate, involved in lipid metabolism were drastically reduced in *phyAB, phyA* and *phyB* mutants, while putrescine and spermidine, involved in polyamine metabolism, were only decreased in *phyB* mutant in contrast to WT in C1 (FL1-H) (Supplementary Table 6).

In C2 (FL2-H), two amino acids (aspartate and homoserine) were reduced in *phyAB*, and one (homoserine) in *phyB* compared with WT. Glucose and fructose levels were higher in *phyAB* while glucose-1-phosphate was lower in *phyB* than in WT in C2 (FL2-H). Decreases of threonate and dehydroascorbate were observed in *phyAB* and *phyB*, respectively, compared with WT in C2 (FL2-H). Moreover, the level of citrate in *phyAB*, and ethanolamine in *phyB* were significantly lower than in WT in C2 (FL2-H). By contrast, spermidine was up-regulated in *phyA* compared with WT in C2 (FL2-H) (Supplementary Table 6).

In C3 (FL1-L), aspartate was down-regulated in *phyAB* but up-regulated in *phyB*, and glycine was up-regulated in *phyAB* compared with WT. Fructose was higher in *phyA* whereas glucose was higher in *phyB* than in WT in C3 (FL1-L). The amount of threonate was higher in *phyB* while ascorbate and dehydroascorbate were lower in *phyB* than in WT in C3 (FL1-L). Citrate content dropped in *phyAB* while succinate content increased in *phyB* compared with WT in C3 (FL1-L). Sinapic acid content was lower in *phyAB* than in WT in C3 (FL1-L) (Supplementary Table 6).

In C4 (FL2-L), increased amounts of GABA, leucine and tryptophan, reduced amount of threonine and increased amount of glucose were found in *phyB* compared with WT. Furthermore, increased levels of myo-inositol and raffinose and reduced level of citrate were observed in *phyAB* compared with WT in C4 (FL2-L) (Supplementary Table 6).

We next performed a correlation analysis using all data available for all genotypes and growth conditions in order to unravel the metabolic network (Supplementary Table 7). Starch amounts were positively correlated with threonate and succinate (Supplementary Table 7). Besides, a large number of amino acids correlated together. Also several lipid and secondary metabolites correlated with one another (Supplementary Table 7).

**FIGURE 4:**
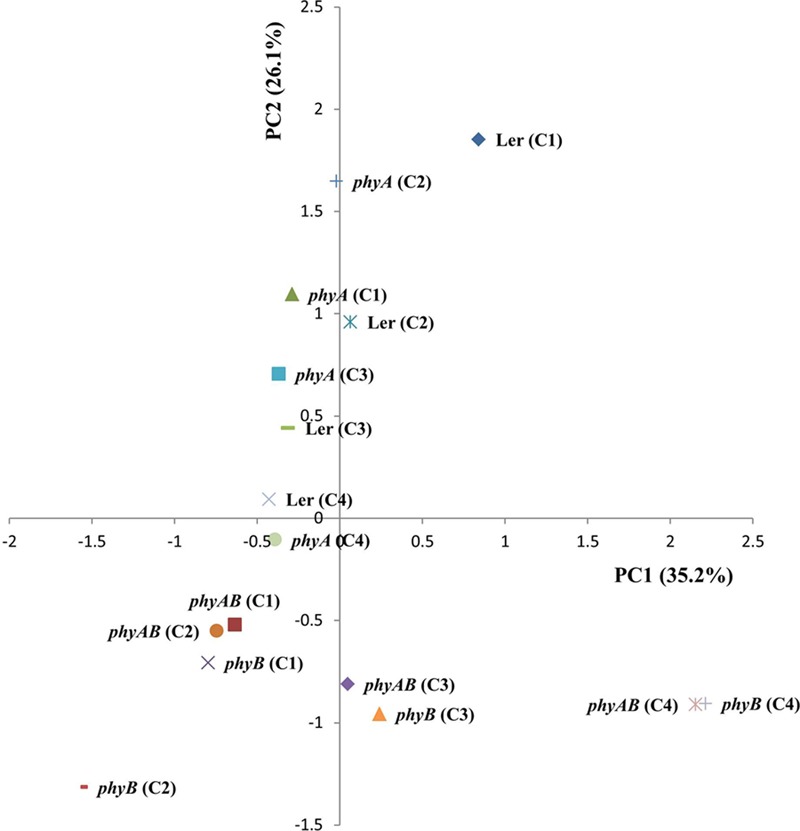
Leaf metabolome analysis of WT and the *phyA, phyB*, and *phyAB* mutants using PCA. Plants were grown in four different white light conditions and sampled at ED.

Next, we performed a principal component analysis (PCA) (**Figure 4**). PC2 separated WT (Ler) and *phyA* from *phyB* and *phyAB*, which suggests that PHYB exerts a major control on primary metabolism. A tendency was also observed for a separation based on light intensity in the first component (PC1) with *phyB* and *phyAB* tending to show an opposite response to light intensity compared to WT and *phyA.* These combined results suggest PHYB may play an important role in the regulation of primary metabolism in response to light intensity.

## Discussion

Photoreceptors regulate many developmental processes in plants (Briggs and Olney, 2001; Millar, 2004). In this study, we investigated the role of photoreceptors in the regulation of primary metabolism, with a particular emphasis on starch accumulation. *cry1* and *cry2* single mutants did not show any significant difference in starch accumulation from WT (**Figure 1**), and also did not show impaired photosystem II (Supplementary Table 3) and retarded growth phenotype (Supplementary Figure 1). By contrast, *hy1/cry1, hy1/cry2* and *hy1/cry1/cry2* all exhibited decreased starch accumulation, being invariant from the *hy1* single mutant (**Figure 1**). Thus it is likely that the HY1 mutation is responsible for the decrease in starch accumulation observed in all these mutants. Moreover, the knockout of HY1, which is characterized by largely dysfunctional PHYs (Muramoto et al., 1999), didn’t show reduced photosynthetic performance (Supplementary Table 3) but exhibited a wide array of other phenotypes such as severely impaired growth, altered leaf structure, modified chloroplast size, and ultrastructure (**Table 2** and Supplementary Figure 2). It was therefore difficult to conclude if the decrease in starch content observed was due to a direct effect on starch metabolism and/or major developmental defects. By contrast, the *phyAB* mutant neither displayed growth (Supplementary Figure 1) and photosynthesis impairment (Supplementary Table 3), nor any aberrant leaf structure phenotype and damaged chloroplast organelles except decreased plastoglobule numbers (**Table 2** and Supplementary Figure 2), but exhibited a decrease in the amount of starch accumulated at ED (**Figure 1**). Thus we decided to avoid *hy1* mutant and focus on *phyAB* mutant. The rationale was that *phyAB* showed a starch phenotype but displayed less other phenotypes under our growth conditions which could have led to confounding pleiotropic effects.

Plastoglobules are lipo-protein particles in chloroplast whose number tend to increase in response to oxidative stress and during senescence (Austin et al., 2006). The observation of reduced number of plastoglobules in *phyAB* in this study (**Table 2** and Supplementary Figure 2) is consistent with the recent report about the involvement of PHYA and PHYB in stress responses (Cerrudo et al., 2012; Gonzalez et al., 2012; Rusaczonek et al., 2015).

The *ndpk2* mutant acts downstream of PHYs and shows defects of cotyledon opening and greening in response to red light (Choi et al., 1999). A deficit in starch accumulation was observed in the *ndpk2* mutant in this study (**Table 3**), suggesting that the regulation of starch metabolism by PHYAB might be transmitted via this protein. Considering the specific sub-cellular localization of NDPK2 in chloroplast (Bayer et al., 2012), we speculate that the regulation of starch accumulation exerted by NDPK2 might occur via post-translational modifications of some starch synthetic enzymes that have previously been identified as subjected to phosphorylation in *Arabidopsis* such as AGPase and starch synthase 3 (Kotting et al., 2010). If so, this regulatory cascade comprising PHYAB, NDPK2 and starch synthetic enzymes would allow plants to control starch synthesis under fluctuating light conditions in addition to the ferredoxin-thioredoxin reductase (FTR)/thioredoxin (Trx) pathway (Thormahlen et al., 2013). A recent study (Chen et al., 2014) identified a wide range of genes regulated by PHYA in *Arabidopsis* seedlings exposed to 3 h of far-red irradiation, and among them several genes involved in starch metabolism were PHYA-induced including *APS1, APL1, SS4, SBE2* (starch branching enzyme II) and *ISA2* (isoamylase II). In this study, we observed a decrease in *APS1* transcripts in *phyAB* compared to WT when plants were grown under white light with PPFD around 115 ± 5 μmol m^-2^ s^-1^ (Supplementary Figure 3), suggesting that PHYAB can induce *APS1* expression in response to both far-red and white light. *APS1* transcript levels were only lower at one time-point (ZT8) over the day course in *phyAB* than WT, suggesting that the regulation of *APS1* by PHYAB is dynamic and might be subject to the control of circadian clock.

We then grew single *phyA* and *phyB* mutants together with the double *phyAB* mutant under red light and white light conditions with different light intensities and light spectral contents. Both *phyA* and *phyB* displayed intermediate starch phenotype between WT and *phyAB* mutant when they were grown in presence of red light (PPFD: 44 ± 1 μmol m^-2^ s^-1^, R:FR: 14.8, PSS: 0.870) (**Figure 3A**), implying both PHYA and PHYB contribute to the regulation of starch accumulation in this condition. Under white light conditions of C2 and C4, the *phyA* mutant showed the same degree of decrease in starch content at ED with *phyAB* compared with WT, and *phyB* had no starch phenotype (**Figure 3B**). This suggests PHYA but not PHYB has a control over starch accumulation in these conditions. Under C1 and C3, neither *phyB* nor *phyA* showed a starch phenotype (**Figure 3B**). However, *phyAB* displayed a decrease in starch accumulation compared with WT (**Figure 3B**), indicating that PHYA and PHYB have complementary roles in the regulation of starch accumulation in these conditions.

The difference between those conditions lies in their spectral content and light intensity: C2 and C4 have higher R:FR and PSS than C1 and C3, and these four light white light treatments have higher light intensity and contrasting R:FR compared to Red light. Therefore, these results suggest PHYA may participate in the regulation of starch accumulation in all the examined light conditions, but PHYB may only have a role under relatively lower R:FR (11.8 ± 0.6) and PSS (0.855 ± 0.001) or low light intensity (44 ± 1 μmol m^-2^ s^-1^). The starch phenotype in *phyAB* in this study is opposite to the higher starch accumulation at ED observed in *phyABDE* compared to WT (Yang et al., 2016). This discrepancy strongly suggests that PHYD and/or PHYE may have roles in inhibiting starch accumulation. Besides, the starch content at EN in *phyAB* is lower than WT in this study under white light (**Figures 1, 2**), which differs from Yang et al. (2016) who find an identical starch content at EN in *phyABDE* and WT. Because the starch degradation rate is influenced by starch ED and also the length of night (Scialdone et al., 2013; Sulpice et al., 2014), the inconsistency of starch contents at EN between both studies might be related to both factors. Indeed, the starch content at ED is different between our study and Yang et al. (2016) and a 16 h light/8 h dark cycle was used in this study whereas Yang et al. (2016) grew their plants in a 12 h light/12 h dark photoperiod.

Interestingly, under red light (PPFD: 44 ± 1 μmol m^-2^ s^-1^), both *phyAB* and *phyB*, but not *phyA* over-accumulated sucrose and glucose (**Figure 3A**), in agreement with (Yang et al., 2016) where the authors observed the same phenotype for *phyBD* and *phyABDE* grown under white light at low fluence (100 μmol m^-2^ s^-1^). Under white light, we did not observe any change in sucrose content (**Figure 1** and Supplementary Table 6). Moderate increases in glucose were only observed in *phyB* at PPFD of 136 ± 3 μmol m^-2^ s^-1^ in both spectral conditions (FL1 and FL2) (Supplementary Table 6). Thus PHYB might have a role in the regulation of sugar metabolism at low light intensities. This could explain the over accumulation of sucrose and glucose observed by Yang et al. (2016) as they grew their plants at a PPFD of 100 μmol m^-2^s^-1^.

Phytochrome A and Phytochrome B did not regulate solely major carbohydrate metabolism. Our metabolic study also revealed that PHYA and PHYB affect a wide range of primary metabolites, in a coordinated manner (Supplementary Table 7), as previously observed for other PHY mutants (Yang et al., 2016). However, in contrast to Yang et al. (2016), the reorganization of the metabolic network did not lead to a striking growth phenotype in this study (Supplementary Figure 1). The PCA analysis in this study suggests PHYB but not PHYA exerts the major control over the leaf metabolic states of plants grown across the four white light conditions with PPFD beyond 136 ± 3 μmol m^-2^ s^-1^ (**Figure 4**), in agreement to a number of previous studies which showed a predominant role of PHYB in the regulation of developmental events when plants were grown under relatively high light intensity. For example, *phyB* showed an equivalent impairment in its stomata development to *phyAB* while *phyA* displayed no effect under a 250 μmol m^-2^ s^-1^ of white light (Boccalandro et al., 2009). Inhibition of hypocotyl elongation was severely impaired in *phyAB* but not in *phyB* under less than 50 μmol m^-2^ s^-1^ of red light, but an intermediate phenotype of hypocotyl elongation between WT and *phyAB* appeared in *phyB* when red light irradiance was elevated (Franklin et al., 2007). Moreover, *phyB* exhibits exclusive enhanced hyponasty compared with WT and *phyA* under high irradiance of 200 μmol m^-2^ s^-1^ (Trupkin et al., 2014). Besides, PCA analysis in this study indicates light intensity also has an impact on the metabolic states (**Figure 4**), which is consistent with the alteration of a large number of metabolites in leaves of plants exposed to different light intensities (Florez-Sarasa et al., 2012).

## Conclusion

PHYAB is essential for a proper control of starch accumulation. The signaling cascade might involve the plastidial NDPK2 and APS1. PHYA likely promotes starch accumulation across a wide range of light conditions while PHYB tends to have a role in relatively low R:FR (11.8 ± 0.6) and PSS (0.855 ± 0.001) or low light intensity (44 ± 1 μmol m^-2^ s^-1^). PHYB has major control on the overall primary metabolic status of plants.

## Author Contributions

RS and XH conceived and designed the study; XH participated in the whole study, TT and AF conducted the GC-MS-based metabolite profiling, PL and PD helped with the whole microscopy study, ND helped with light spectra analysis, and AE-F helped with laboratory work; XH and RS wrote the manuscript. All authors reviewed the manuscript and agreed on its content.

## Conflict of Interest Statement

The authors declare that the research was conducted in the absence of any commercial or financial relationships that could be construed as a potential conflict of interest.

## References

[B1] Ahmad M. Cashmore A. R. (1993). *Hy4* gene of *A. thaliana* encodes a protein with characteristics of a blue-light photoreceptor. *Nature* 366 162–166. 10.1038/366162a0 8232555

[B2] Ahmad M. Jarillo J. A. Cashmore A. R. (1998). Chimeric proteins between cry1 and cry2 Arabidopsis blue light photoreceptors indicate overlapping functions and varying protein stability. *Plant Cell* 10 197–207. 10.2307/3870698 9490743 PMC143983

[B3] Ahmad M. Lin C. T. Cashmore A. R. (1995). Mutations throughout an *Arabidopsis* blue-light photoreceptor impair blue-light-responsive anthocyanin accumulation and inhibition of hypocotyl elongation. *Plant J.* 8 653–658. 10.1046/j.1365-313X.1995.08050653.x 8528277

[B4] Austin J. R. Frost E. Vidi P. A. Kessler F. Staehelin L. A. (2006). Plastoglobules are lipoprotein subcompartments of the chloroplast that are permanently coupled to thylakoid membranes and contain biosynthetic enzymes. *Plant Cell* 18 1693–1703. 10.1105/tpc.105.039859 16731586 PMC1488921

[B5] Barneche F. Winter V. Crevecoeur M. Rochaix J. D. (2006). ATAB2 is a novel factor in the signalling pathway of light-controlled synthesis of photosystem proteins. *EMBO J.* 25 5907–5918. 10.1038/sj.emboj.7601472 17139246 PMC1698907

[B6] Bayer R. G. Stael S. Rocha A. G. Mair A. Vothknecht U. C. Teige M. (2012). Chloroplast-localized protein kinases: a step forward towards a complete inventory. *J. Exp. Bot.* 63 1713–1723. 10.1093/jxb/err377 22282538 PMC3971369

[B7] Boccalandro H. E. Rugnone M. L. Moreno J. E. Ploschuk E. L. Serna L. Yanovsky M. J. (2009). Phytochrome B enhances photosynthesis at the expense of water-use efficiency in Arabidopsis. *Plant Physiol.* 150 1083–1092. 10.1104/pp.109.135509 19363093 PMC2689964

[B8] Briggs W. R. Olney M. A. (2001). Photoreceptors in plant photomorphogenesis to date. Five phytochromes, two cryptochromes, one phototropin, and one superchrome. *Plant Physiol.* 125 85–88. 10.1104/Pp.125.1.85 11154303 PMC1539332

[B9] Cashmore A. R. Jarillo J. A. Wu Y. J. Liu D. M. (1999). Cryptochromes: blue light receptors for plants and animals. *Science* 284 760–765. 10.1126/science.284.5415.760 10221900

[B10] Castillon A. Shen H. Huq E. (2007). Phytochrome Interacting Factors: central players in phytochrome-mediated light signaling networks. *Trends Plant Sci.* 12 514–521. 10.1016/j.tplants.2007.10.001 17933576

[B11] Castillon A. Shen H. Huq E. (2009). Blue light induces degradation of the negative regulator phytochrome interacting factor 1 to promote photomorphogenic development of Arabidopsis seedlings. *Genetics* 182 161–171. 10.1534/genetics.108.099887 19255368 PMC2674814

[B12] Cerrudo I. Keller M. M. Cargnel M. D. Demkura P. V. de Wit M. Patitucci M. S. (2012). Low Red/Far-red ratios reduce Arabidopsis resistance to *Botrytis cinerea* and jasmonate responses via a COI1-JAZ10-dependent, salicylic acid-independent mechanism. *Plant Physiol.* 158 2042–2052. 10.1104/pp.112.193359 22371506 PMC3320205

[B13] Chen F. Li B. S. Li G. Charron J. B. Dai M. Q. Shi X. R. (2014). *Arabidopsis* phytochrome A directly targets numerous promoters for individualized modulation of genes in a wide range of pathways. *Plant Cell* 26 1949–1966. 10.1105/tpc.114.123950 24794133 PMC4079361

[B14] Choi G. Yi H. Lee J. Kwon Y. K. Soh M. S. Shin B. C. (1999). Phytochrome signalling is mediated through nucleoside diphosphate kinase 2. *Nature* 401 610–613. 10.1038/44176 10524631

[B15] Chory J. Peto C. A. Ashbaugh M. Saganich R. Pratt L. Ausubel F. (1989). Different roles for phytochrome in etiolated and green plants deduced from characterization of *Arabidopsis thaliana* mutants. *Plant Cell* 1 867–880. 10.1105/tpc.1.9.867 12359912 PMC159823

[B16] Chun L. Kawakami A. Christopher D. A. (2001). Phytochrome A mediates blue light and UV-A-dependent chloroplast gene transcription in green leaves. *Plant Physiol.* 125 1957–1966. 10.1104/pp.125.4.1957 11299375 PMC88851

[B17] Clack T. Mathews S. Sharrock R. A. (1994). The phytochrome apoprotein family in *Arabidopsis* is encoded by 5 genes - the sequences and expression of *PHYD* and *PHYE*. *Plant Mol. Biol.* 25 413–427. 10.1007/Bf00043870 8049367

[B18] Clough R. C. Vierstra R. D. (1997). Phytochrome degradation. *Plant Cell Environ.* 20 713–721. 10.1046/j.1365-3040.1997.d01-107.x

[B19] Cross J. M. von Korff M. Altmann T. Bartzetko L. Sulpice R. Gibon Y. (2006). Variation of enzyme activities and metabolite levels in 24 arabidopsis accessions growing in carbon-limited conditions. *Plant Physiol.* 142 1574–1588. 10.1104/pp.106.086629 17085515 PMC1676042

[B20] Darko E. Heydarizadeh P. Schoefs B. Sabzalian M. R. (2014). Photosynthesis under artificial light: the shift in primary and secondary metabolism. *Philos. Trans. R. Soc. B Biol. Sci.* 369 20130243 10.1098/Rstb.2013.0243 PMC3949401 24591723

[B21] Fankhauser C. Staiger D. (2002). Photoreceptors in *Arabidopsis thaliana*: light perception, signal transduction and entrainment of the endogenous clock. *Planta* 216 1–16. 10.1007/s00425-002-0831-4 12430009

[B22] Florez-Sarasa I. Araujo W. L. Wallstrom S. V. Rasmusson A. G. Fernie A. R. Ribas-Carbo M. (2012). Light-responsive metabolite and transcript levels are maintained following a dark-adaptation period in leaves of *Arabidopsis thaliana*. *New Phytol.* 195 136–148. 10.1111/j.1469-8137.2012.04153.x 22548389

[B23] Franklin K. A. Allen T. Whitelam G. C. (2007). Phytochrome A is an irradiance-dependent red light sensor. *Plant J.* 50 108–117. 10.1111/j.1365-313X.2007.03036.x 17346261

[B24] Franklin K. A. Davis S. J. Stoddart W. M. Vierstra R. D. Whitelam G. C. (2003). Mutant analyses define multiple roles for phytochrome C in Arabidopsis photomorphogenesis. *Plant Cell* 15 1981–1989. 10.1105/Tpc.015164 12953105 PMC181325

[B25] Franklin K. A. Quail P. H. (2010). Phytochrome functions in *Arabidopsis* development. *J. Exp. Bot.* 61 11–24. 10.1093/jxb/erp304 19815685 PMC2800801

[B26] Genty B. Briantais J. M. Baker N. R. (1989). The relationship between the quantum yield of photosynthetic electron-transport and quenching of chlorophyll fluorescence. *Biochim. Biophys. Acta* 990 87–92. 10.1016/S0304-4165(89)80016-9

[B27] Genty B. Harbinson J. Cailly A. Rizza F. (1996). Fate of excitation at PS II in leaves: the non-photochemical side. *Paper Presented at The Third BBSRC Robert Hill Symposium on Photosynthesis, March 31 to April 3, 1996* University of Sheffield Sheffield.

[B28] Gonzalez C. V. Ibarra S. E. Piccoli P. N. Botto J. F. Boccalandro H. E. (2012). Phytochrome B increases drought tolerance by enhancing ABA sensitivity in *Arabidopsis thaliana*. *Plant Cell Environ.* 35 1958–1968. 10.1111/j.1365-3040.2012.02529.x 22553988

[B29] Gururani M. A. Mohanta T. K. Bae H. (2015). Current understanding of the interplay between phytohormones and photosynthesis under environmental stress. *Int. J. Mol. Sci.* 16 19055–19085. 10.3390/ijms160819055 26287167 PMC4581286

[B30] Jones A. M. Allen C. D. Gardner G. Quail P. H. (1986). Synthesis of phytochrome apoprotein and chromophore are not coupled obligatorily. *Plant Physiol.* 81 1014–1016. 10.1104/Pp.81.4.1014 16664935 PMC1075477

[B31] Jumtee K. Bamba T. Okazawa A. Fukusaki E. Kobayashi A. (2008). Integrated metabolite and gene expression profiling revealing phytochrome A regulation of polyamine biosynthesis of *Arabidopsis thaliana*. *J. Exp. Bot.* 59 1187–1200. 10.1093/jxb/ern026 18375607

[B32] Kitajima M. Butler W. L. (1975). Quenching of chlorophyll fluorescence and primary photochemistry in chloroplasts by dibromothymoquinone. *Biochim. Biophys. Acta* 376 105–115. 10.1016/0005-2728(75)90209-1 1125215

[B33] Kleine T. Lockhart P. Batschauer A. (2003). An *Arabidopsis* protein closely related to *Synechocystis* cryptochrome is targeted to organelles. *Plant J.* 35 93–103. 10.1046/j.1365-313X.2003.01787.x 12834405

[B34] Kotting O. Kossmann J. Zeeman S. C. Lloyd J. R. (2010). Regulation of starch metabolism: the age of enlightenment? *Curr. Opin. Plant Biol.* 13 321–329. 10.1016/j.pbi.2010.01.003 20171927

[B35] Lagarias J. C. Rapoport H. (1980). Chromopeptides from phytochrome - the structure and linkage of the pr form of the phytochrome chromophore. *J. Am. Chem. Soc.* 102 4821–4828. 10.1021/Ja00534a042

[B36] Lin C. T. Yang H. Y. Guo H. W. Mockler T. Chen J. Cashmore A. R. (1998). Enhancement of blue-light sensitivity of *Arabidopsis* seedlings by a blue light receptor cryptochrome 2. *Proc. Natl. Acad. Sci. U.S.A.* 95 2686–2690. 10.1073/pnas.95.5.2686 9482948 PMC19462

[B37] Lisec J. Schauer N. Kopka J. Willmitzer L. Fernie A. R. (2006). Gas chromatography mass spectrometry-based metabolite profiling in plants. *Nat. Protoc.* 1 387–396. 10.1038/nprot.2006.59 17406261

[B38] Mao J. Zhang Y. C. Sang Y. Li Q. H. Yang H. Q. (2005). A role for *Arabidopsis* cryptochromes and COP1 in the regulation of stomatal opening. *Proc. Natl. Acad. Sci. U.S.A.* 102 12270–12275. 10.1073/pnas.0501011102 16093319 PMC1189306

[B39] Millar A. J. (2004). Input signals to the plant circadian clock. *J. Exp. Bot.* 55 277–283. 10.1093/jxb/erh034 14695902

[B40] Moglich A. Yang X. J. Ayers R. A. Moffat K. (2010). Structure and function of plant photoreceptors. *Annu. Rev. Plant Biol.* 61 21–47. 10.1146/annurev-arplant-042809-112259 20192744

[B41] Mugford S. T. Fernandez O. Brinton J. Flis A. Krohn N. Encke B. (2014). Regulatory properties of ADP glucose pyrophosphorylase are required for adjustment of leaf starch synthesis in different photoperiods. *Plant Physiol.* 166 1733–U1877. 10.1104/pp.114.247759 25293961 PMC4256850

[B42] Muramoto T. Kohchi T. Yokota A. Hwang I. H. Goodman H. M. (1999). The Arabidopsis photomorphogenic mutant *hy1* is deficient in phytochrome chromophore biosynthesis as a result of a mutation in a plastid heme oxygenase. *Plant Cell* 11 335–347. 10.1105/tpc.11.3.335 10072395 PMC144190

[B43] Parks B. M. Quail P. H. (1993). *Hy8*, a new class of *Arabidopsis* long hypocotyl mutants deficient in functional phytochrome A. *Plant Cell* 5 39–48. 10.1105/tpc.5.1.39 8439743 PMC160248

[B44] Pyl E. T. Piques M. Ivakov A. Schulze W. Ishihara H. Stitt M. (2012). Metabolism and growth in *Arabidopsis* depend on the daytime temperature but are temperature-compensated against cool nights. *Plant Cell* 24 2443–2469. 10.1105/tpc.112.097188 22739829 PMC3406903

[B45] Reed J. W. Nagatani A. Elich T. D. Fagan M. Chory J. (1994). Phytochrome A and phytochrome B have overlapping but distinct functions in Arabidopsis development. *Plant Physiol.* 104 1139–1149. 10.1104/pp.104.4.1139 12232154 PMC159274

[B46] Reed J. W. Nagpal P. Poole D. S. Furuya M. Chory J. (1993). Mutations in the gene for the red/far-red light receptor phytochrome B alter cell elongation and physiological responses throughout Arabidopsis development. *Plant Cell* 5 147–157. 10.1105/Tpc.5.2.147 8453299 PMC160258

[B47] Rusaczonek A. Czarnocka W. Kacprzak S. Witon D. Slesak I. Szechynska-Hebda M. (2015). Role of phytochromes A and B in the regulation of cell death and acclimatory responses to UV stress in *Arabidopsis thaliana*. *J. Exp. Bot.* 66 6679–6695. 10.1093/jxb/erv375 26385378 PMC4623682

[B48] Saebo A. Krekling T. Appelgren M. (1995). Light quality affects photosynthesis and leaf anatomy of birch plantlets *in vitro*. *Plant Cell Tissue Organ Cult.* 41 177–185. 10.1007/BF00051588

[B49] Sager J. C. Smith W. O. Edwards J. L. Cyr K. L. (1988). Photosynthetic efficiency and phytochrome photoequilibria determination using spectral data. *Trans. ASAE* 31 1882–1889. 10.13031/2013.30952

[B50] Scialdone A. Mugford S. T. Feike D. Skeffington A. Borrill P. Graf A. (2013). Arabidopsis plants perform arithmetic division to prevent starvation at night. *Elife* 2:e00669 10.7554/eLife.00669 PMC3691572 23805380

[B51] Sulpice R. Flis A. Ivakov A. A. Apelt F. Krohn N. Encke B. (2014). *Arabidopsis* coordinates the diurnal regulation of carbon allocation and growth across a wide range of photoperiods. *Mol. Plant* 7 137–155. 10.1093/mp/sst127 24121291

[B52] Sulpice R. Pyl E. T. Ishihara H. Trenkamp S. Steinfath M. Witucka-Wall H. (2009). Starch as a major integrator in the regulation of plant growth. *Proc. Natl. Acad. Sci. U.S.A.* 106 10348–10353. 10.1073/pnas.0903478106 19506259 PMC2693182

[B53] Tenorio G. Orea A. Romero J. M. Merida A. (2003). Oscillation of mRNA level and activity of granule-bound starch synthase I in Arabidopsis leaves during the day/night cycle. *Plant Mol. Biol.* 51 949–958. 10.1023/A:1023053420632 12777053

[B54] Thormahlen I. Ruber J. Von Roepenack-Lahaye E. Ehrlich S. M. Massot V. Hummer C. (2013). Inactivation of thioredoxin *f1* leads to decreased light activation of ADP-glucose pyrophosphorylase and altered diurnal starch turnover in leaves of *Arabidopsis* plants. *Plant Cell Environ.* 36 16–29. 10.1111/j.1365-3040.2012.02549.x 22646759

[B55] Toledo-Ortiz G. Johansson H. Lee K. P. Bou-Torrent J. Stewart K. Steel G. (2014). The HY5-PIF regulatory module coordinates light and temperature control of photosynthetic gene transcription. *PLoS Genet.* 10:e1004416 10.1371/journal.pgen.1004416 PMC4055456 24922306

[B56] Trupkin S. A. Legris M. Buchovsky A. S. Rivero M. B. T. Casal J. J. (2014). Phytochrome B nuclear bodies respond to the low red to far-red ratio and to the reduced irradiance of canopy shade in Arabidopsis. *Plant Physiol.* 165 1698–1708. 10.1104/pp.114.242438 24948827 PMC4119049

[B57] Usami T. Mochizuki N. Kondo M. Nishimura M. Nagatani A. (2004). Cryptochromes and phytochromes synergistically regulate *Arabidopsis* root greening under blue light. *Plant Cell Physiol.* 45 1798–1808. 10.1093/pcp/pch205 15653798

[B58] Yang D. Y. Seaton D. D. Krahmer J. Halliday K. J. (2016). Photoreceptor effects on plant biomass, resource allocation, and metabolic state. *Proc. Natl. Acad. Sci. U.S.A.* 113 7667–7672. 10.1073/pnas.1601309113 27330114 PMC4941476

[B59] Yano S. Terashima I. (2001). Separate localization of light signal perception for sun or shade type chloroplast and palisade tissue differentiation in *Chenopodium album*. *Plant Cell Physiol.* 42 1303–1310. 10.1093/Pcp/Pce183 11773522

[B60] Yu X. Liu H. Klejnot J. Lin C. (2010). The cryptochrome blue light receptors. *Arabidopsis Book* 8:e0135 10.1199/tab.0135 PMC3155252 21841916

