# Supplementary material for: Phytochrome A and B Regulate Primary Metabolism in Arabidopsis Leaves in Response to Light
Source: Front Plant Sci. 2017 Aug 9;8:1394. doi: 10.3389/fpls.2017.01394 (PMC5552712; doi:10.3389/fpls.2017.01394)
Supplement: Supplementary file 1 [file Presentation_1.PDF]

Phytochrome A and B regulate primary metabolism in *Arabidopsis* leaves in response to light

Author: Xiaozhen Han[1], Takayuki Tohge[2], Pierce Lalor[3], Peter Dockery[3], Nicholas Devaney[4], Alberto Abrantes Esteves-Ferreira [1], Alisdair R. Fernie[2], Ronan Sulpice[1]*

1. Plant Systems Biology Research Lab, Plant and AgriBiosciences, Aras de Brun, National University of Ireland, Galway, Ireland
2. Max Planck Institute of Molecular Plant Physiology, Potsdam-Golm, Germany
3. Centre for Microscopy and Imaging, Anatomy, School of Medicine, NUI Galway, Galway, Ireland
4. School of Physics, National University of Ireland, Galway, Ireland

Correspondence:

Dr Ronan Sulpice

Ronan.sulpice@nuigalway.ie

Supplementary Table 1 Information about the mutant lines

Mutant	AGI code	Background	NASC ID	Seed name (Allele)
cry2	At1g04400	Col-4	N3732	cry2-1
cry1	At4g08920	Col-0	N662234	SALK_042397C
phyAB	At1g09570	Ler	N6224	phyA-201
	At2g18790			phyB-5
phyA	At1g09570	Ler	N6219	phyA-201
phyB	At2g18790	Ler	N69	phyB-1
hy1	At2g26670	Ler	NW67	hy1-1
hy1/cry1	At4g08920	Ler	N9855	cry1
	At2g26670			hy1
hy1/cry2	At1g04400	Ler	N9856	cry2
	At2g26670			hy1
hy1/cry1/cry2	At4g08920	Ler	N9854	hy1
	At1g04400			cry1
	At2g26670			cry2
ndpk2	At5g63310	Col-0	N823418	SAIL_553_F09

Supplementary Table 2 Sequence (5'→3') of the primers used for the Q-PCR experiments

	AGI code	Forward primer	Reverse primer
GBSS1	AT1G32900	GGATCCAGATGATGTGATAGCAACAGC	CCTCAACCTTCCCTACTCCTAACTTCCC
APL1	AT5G19220	GTTCCCATTGGAATAGGAGAGAACAC	GACCTATCTGCTTCTTGTATTCCCTC
APS1	AT5G48300	CTACACACAGCCGCGTTATTTACCAC	TATGCAGGAACGGAGTCCAACCACAG
SS1	AT5G24300	GGAACCATTCCAGTTGTTCATGGCAC	TTTCGACAAGGGAGTGAAGACCCACC
SS2	AT3G01180	TGAAACGGGTCTTGGTTGGACATTTG	TTCTCTGAAGCCCTTCCCAACTCTCC
SS4	AT4G18240	GAGGCTTAAATGACAGTGTCTTCGAC	CCAACGCATAATTGAAACCCTGTTCG

A

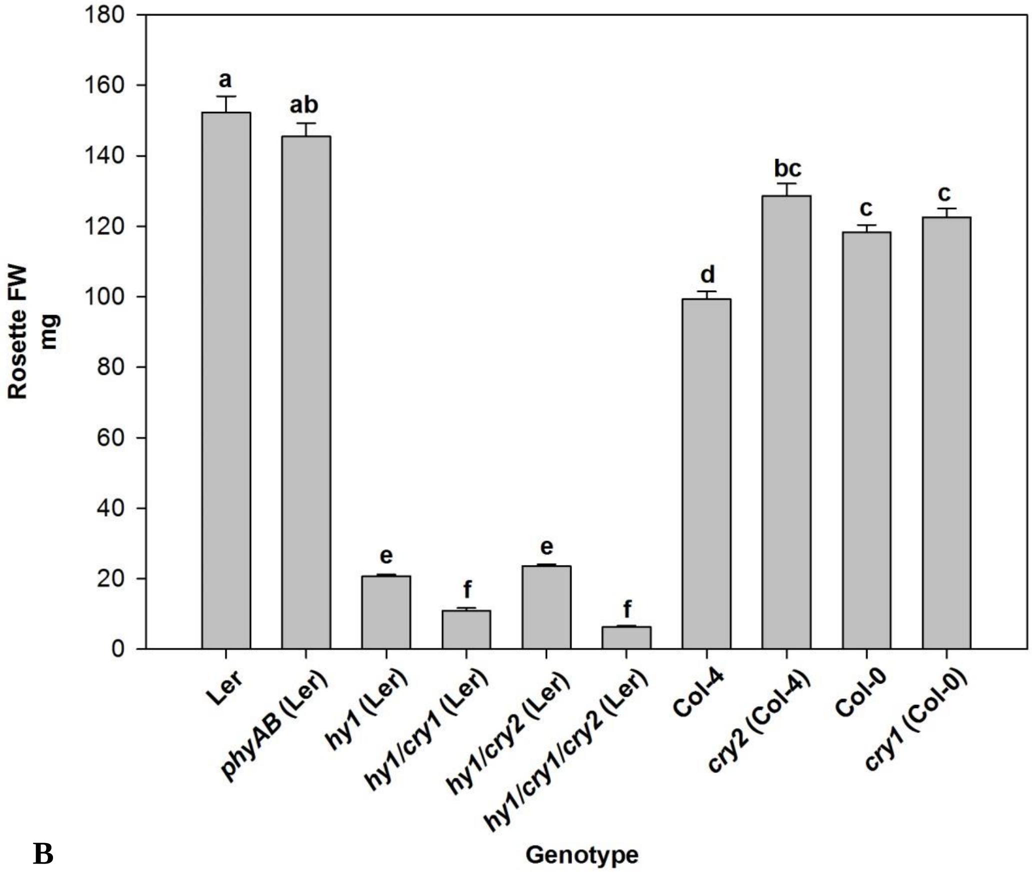

B

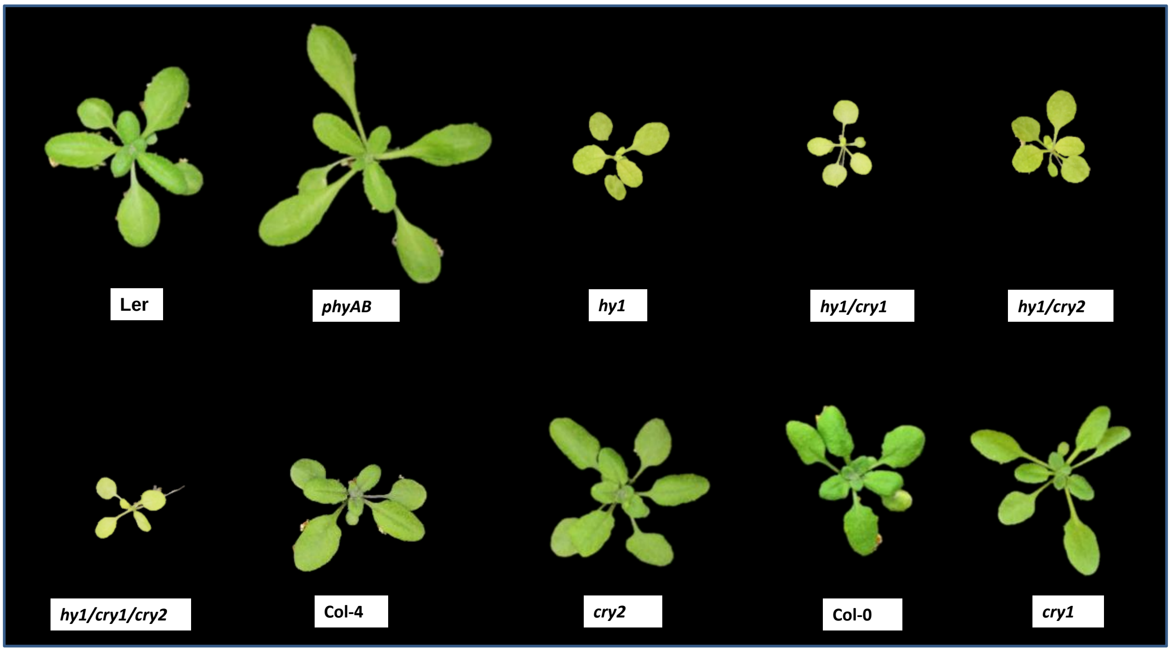

Supplementary Figure 1. A: Fresh biomass of photoreceptor mutants and their respective wild types. The background ecotypes for the mutants are indicated in the brackets. Values are means ± SE of measurements made on ten rosettes; B: Pictures taken before harvesting. Plants were grown under FL2 with PPFD of 115±5 μmol m^{-2} s^{-1}.

Supplementary Table 3 Chlorophyll fluorescence parameters of photoreceptor mutants and their respective wild types

	F_v/F_m	Y(II)	Y(NPQ)	Y(NO)
Ler	0.798±0.005ab	0.387±0.008b	0.313±0.005c	0.300±0.003ab
phyAB (Ler)	0.788±0.003bc	0.392±0.006b	0.312±0.005c	0.296±0.007ab
hy1 (Ler)	0.798±0.001ab	0.392±0.009b	0.296±0.018cd	0.303±0.014ab
hy1/cry1 (Ler)	0.787±0.002c	0.266±0.012c	0.37±0.017ab	0.340±0.015a
hy1/cry2 (Ler)	0.802±0.001ab	0.391±0.005b	0.330±0.012bc	0.278±0.008b
hy1/cry1/cry2 (Ler)	0.785±0.001c	0.267±0.009c	0.373±0.017a	0.357±0.014a
Col-4	0.793±0.001ab	0.448±0.009a	0.248±0.011de	0.304±0.003ab
cry2 (Col-4)	0.803±0.002a	0.455±0.013a	0.246±0.012de	0.299±0.012ab
Col-0	0.789±0.005bc	0.491±0.004a	0.195±0.004e	0.314±0.004ab
cry1 (Col-0)	0.794±0.001ab	0.453±0.008a	0.238±0.012de	0.310±0.009ab

Plants were grown under FL2 with PPFD of 115±5 μmol m^{-2} s^{-1}. Results are mean ±SE of independent measurements on four individual plants for each genotype. The background ecotype for each mutant is indicated in the bracket.

Supplementary Table 4 Glucose levels at ED and EN in photoreceptor mutants and their respective wild types

	Glucose at ED (μmol eq. glc g^{-1} FW)	Glucose at EN (μmol eq. glc g^{-1} FW)
Ler	0.20±0.02b	0.21±0.02a
phyAB (Ler)	0.34±0.03a	0.24±0.08a
hy1 (Ler)	0.15±0.01b	0.20±0.02a
hy1/cry1 (Ler)	0.13±0.02b	0.17±0.01a
hy1/cry2 (Ler)	0.16±0.00b	0.28±0.08a
hy1/cry1/cry2 (Ler)	0.15±0.02b	0.17±0.04a
Col-4	0.32±0.02a	0.22±0.02a
Cry2 (Col-4)	0.26±0.02ab	0.35±0.05a
Col-0	0.28±0.01ab	0.25±0.02a
Cry1 (Col-0)	0.26±0.01ab	0.26±0.03a

The background ecotype for each mutant is indicated in the brackets. Plants were grown under FL2 with PPFD of 115±5 μmol m^{-2} s^{-1}. Results are mean ±SE of measurements made on five biological samples.

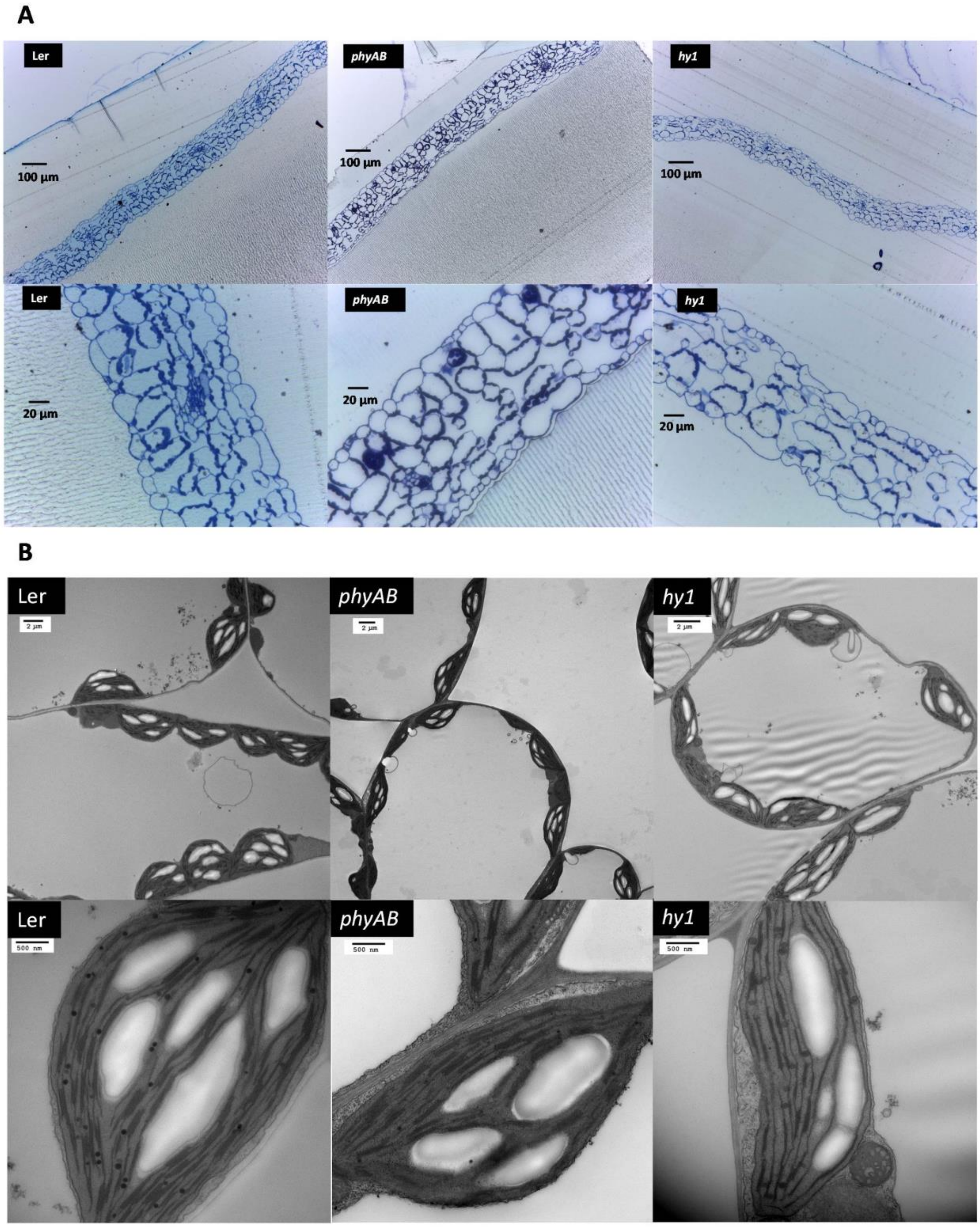

Supplementary Figure 2. Leaf anatomy in Ler, *phyAB* and *hy1*. (A): Light micrographs of leaf transverse sections. (B): Transmission electron micrographs of leaf sections.

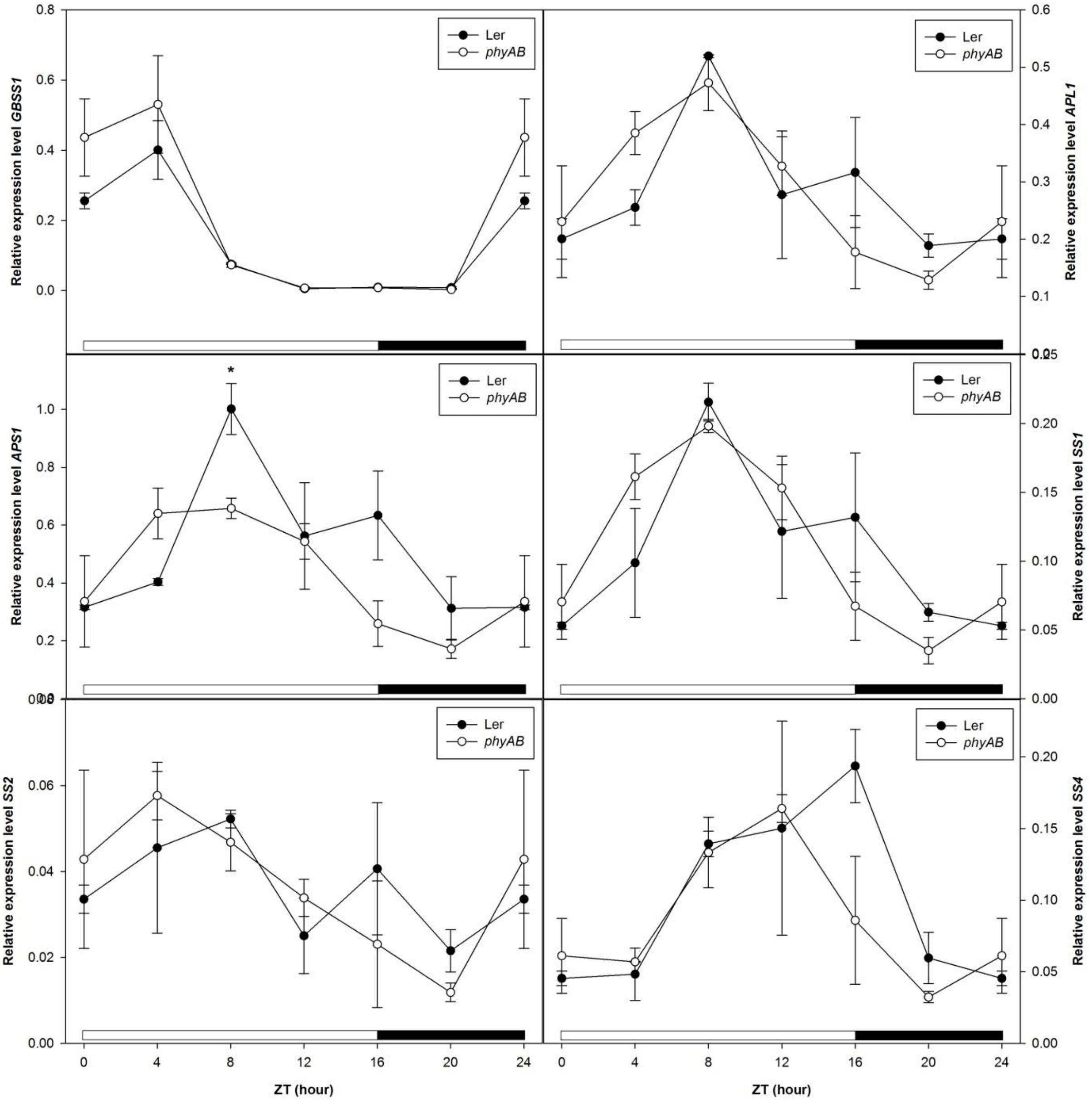

Supplementary Figure 3. Expression profiles of *GBSS1*, *APL1*, *APS1*, *SS1*, *SS2* and *SS4* during a diurnal cycle in *phyAB* and wild type. Plants were grown under FL2 with PPFD of 115±5 µmol m^{-2} s^{-1}. Results are mean ±SE of measurements made on three biological samples (each biological sample contain two rosettes).

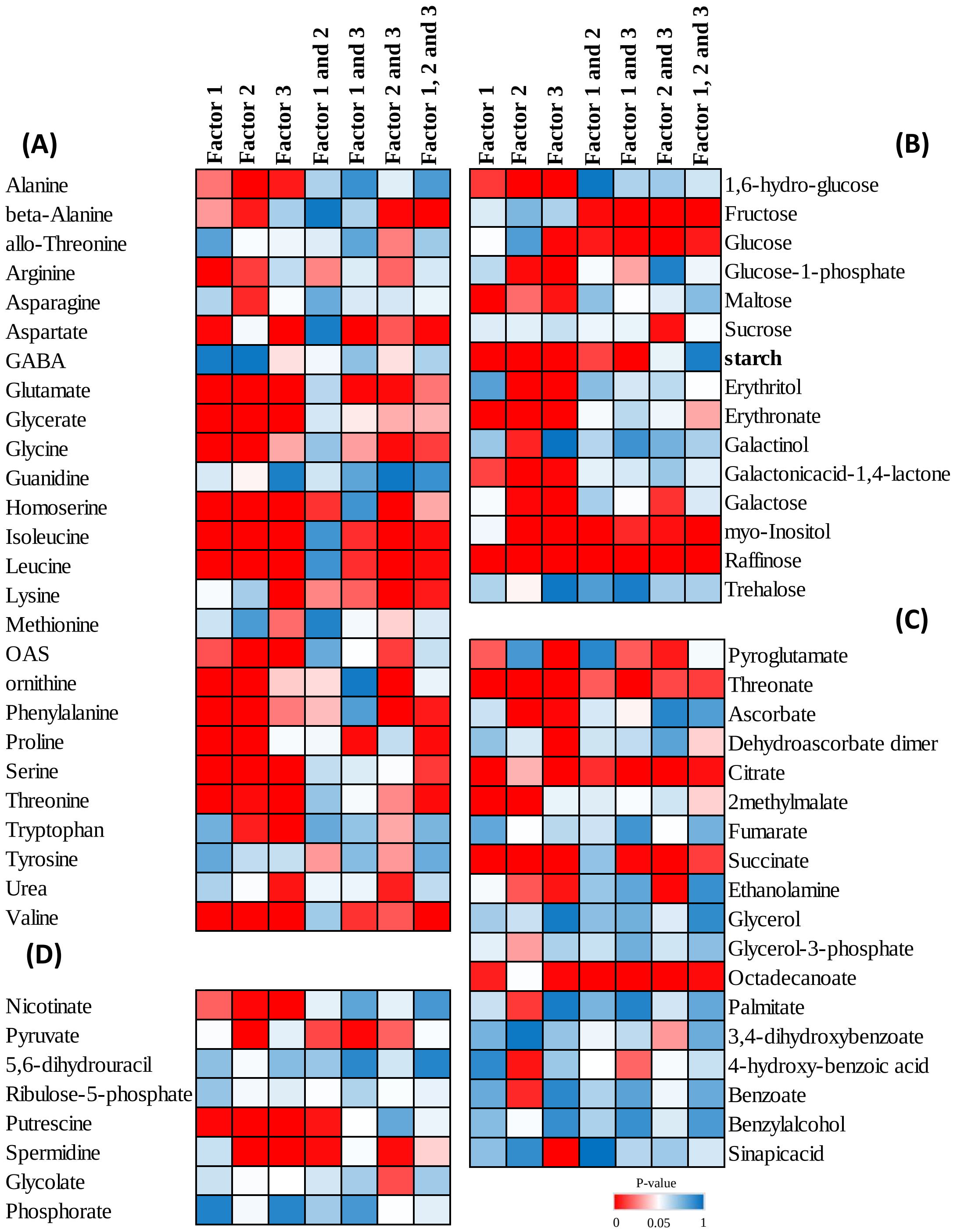

Supplementary Figure 4. Effects of light spectral content (Factor 1: FL1 and FL2), PPFD (Factor 2: High and Low) and genotype (Factor 3: *phyA*, *phyB*, *phyAB* and WT) on the *Arabidopsis* rosette metabolome (Three-way ANOVA). P-values are colored red if they are less than 0.05, and colored blue if they are beyond 0.05. Metabolite profiles were performed on the same samples as in Fig. 3B. A: Metabolites in amino acid metabolism. B: Metabolites in carbohydrate metabolism. C: Metabolites in redox regulation, glyoxylate cycle, lipid metabolism and secondary metabolism. D: Others.
